# Simple One-Pot Syntheses and Characterizations of Free Fluoride- and Bifluoride-Containing Polymers Soluble in Non-Aqueous Solvents

**DOI:** 10.3390/ma9120965

**Published:** 2016-11-30

**Authors:** Dominik Steinle, Laura Friedrich, Nico Bevilacqua, Elizabeth von Hauff, Fabienne Gschwind

**Affiliations:** 1 Helmholtz Institute Ulm (HIU), Helmholtzstrasse 11, 89081 Ulm, Germany; dominik.steinle@uni-ulm.de (D.S.); Laura.Friedrich@uni-ulm.de (L.F.); nico.bevilacqua@uni-ulm.de (N.B.); 2 Department of Physics and Astronomy, VU Amsterdam, De Boelelaan 1081, 1081 HV Amsterdam, The Netherlands; e.l.von.hauff@vu.nl

**Keywords:** fluoride, bifluoride, polymer, conductivity, quantitative measurement, fluoride ion batteries, electrolyte

## Abstract

One of the problems that arise with bifluoride- or fluoride-containing compounds is their poor solubility in non-aqueous solvents. We report herein a facile one-pot synthesis and the chemical analysis of fluoride/bifluoride-containing polymers, which are soluble in MeCN. Different polymers, such as Polyvinylacetate or Polyethylene imine and saccharides, such as maltodextrin, were complexed with ammonium (bi)fluoride using hydrogen bonds to form the desired (bi)fluoride-containing compounds. The newly formed hydrogen bonding (bi)fluoride-doped polymer matrices were analyzed using infrared and nuclear magnetic resonance spectroscopies, and X-ray diffraction. The promising materials also underwent impedance spectroscopy, conductivity measurements and preliminary tests as electrolytes for room temperature fluoride ion batteries along with an analysis of their performance.

## 1. Introduction

The incorporation of fluorine into polymers is widely known, especially those with C–F bonds (the so-called “fluoropolymers”), the most famous of which being Polyvinyl fluoride (PVF), Polyvinyliden fluoride (PVDF), and Polyterafluoroethylene (PTFE). These fluoropolymers all have a wide impact on our daily lives [1], for instance, in household items such as pans or clothing (Teflon^®^, and Goretex^®^), as binders in lithium ion batteries [2], as membranes in fuel cells [3,4], and even in anti-fouling paint for ships [5,6].

Nevertheless, polymers containing free fluoride species, where the fluoride component, (F^−^ or F_2_H^−^) is not covalently bound to the carbon backbone, are very rare. Poly[4-vinylpyridinium poly(hydrogen fluoride)] is one of the few commercially available polymers containing hydrogen fluoride and is used as a fluoridation agent [7,8]. Even so, the free fluoride ion is used widely in the syntheses of organofluoride derivatives; for example, compounds containing free fluorides are used as fluoridation agents [9]. Tetraalkylammonium fluoride has, in particular, been an important reagent in this field for many years, despite its disadvantages like decomposition upon drying [10,11]. Today, more selective and safer materials can be used, e.g., the commercially available “selectfluor” fluoridation agents comprised of diazobicyclo octane [12].

Furthermore, ionic fluoride is also used as a proton abstractor in base-assisted reactions [13] and as a promoter for organosilyl reactions.

To be able to measure the fluoride content of a solution, the so-called fluoride sensing method can be used. In this method, organic molecules bind fluoride ions selectively, and can be detected, for instance, by optical methods [14,15,16]. Additionally, the nature of the free or naked fluoride ion has been discussed in the literature, especially towards its potential to form very strong hydrogen bonds [17,18,19,20]. There are also some reports about the crystal structure of the bifluoride anion and its ability to form strong hydrogen bonds [21]. 

Our work pertains to electrolytes for anion batteries, namely room-temperature fluoride ion batteries [22,23,24], where we are investigating fluoride-containing compounds, which should have the following attributes: (i) solubility in non-aqueous solvents; (ii) fluoride species that are free to migrate and not bonded within a structure; (iii) do not undergo decomposition to form HF; (iv) contain molecules which will not undergo decomposition during electrochemical reactions; and (v) fluoride-containing electrolytes that allow for charge transport between the electrodes.

**Figure 1:**
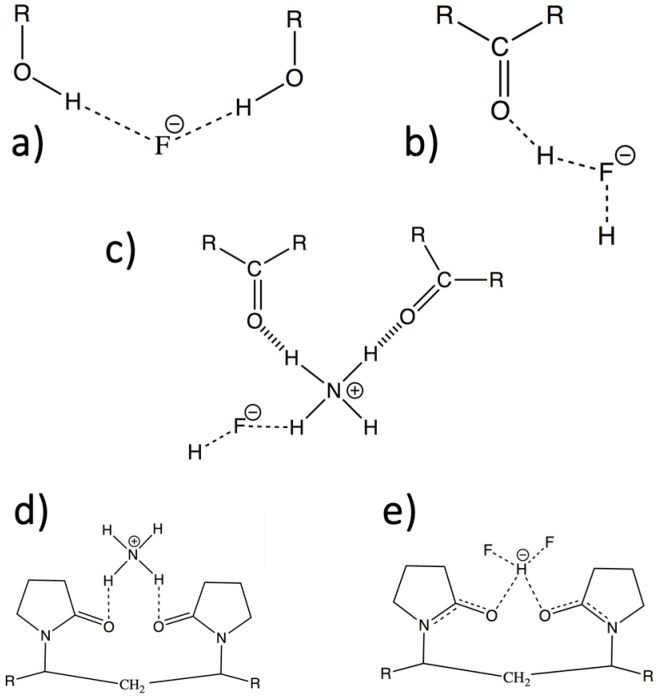
A selection of possible coordination geometries of fluoride ions and ammonium ions to different functional groups: (**a**) Coordination of F^−^ to alcohol groups; (**b**) Coordination of F_2_H^−^ to any kind of C=O; (**c**) Coordination of the ammonium ion to an aldehyde or acetate type of functional group and further coordination to F_2_H^−^; and (**d**,**e**) Example of coordination to polyvinyl pyrrolidone (PVP) with the ammonium group or with the F_2_H^−^ ion.

In the beginning, ionic liquids such as alkyl ammonium fluoride were tested as electrolytes but failed since too many side-processes were ongoing and discharge could not be clearly attributed to the transport of fluoride. We decided therefore to investigate polymer matrices that contained fluoride or bifluoride anions. Until now, only poly(ethylene glycol) (PEG) derivatives have been tested, where the PEG polymer was reacted with ammonium (bi)fluoride in MeCN, with the hypothesis that the ammonium ions would form hydrogen bonds with the ethereal oxygen atom. In this way, a polycation was formed that forces the otherwise insoluble fluoride species to dissolve in the solution [22]. It remains unclear, however, as to the types of bonding interactions that are formed among the bifluoride anion, the ammonium ion and the polymer (Figure 1). Furthermore, the species in solution exist in a complex equilibrium.

It should be noted that these fluoride-doped PEG matrices contained exclusively bifluoride (F_2_H^−^), instead of fluoride (F^−^), as its anionic species. This result is due to the strong proton-abstracting power of fluoride ions, which can abstract protons from either the polymer or from the solvent itself. 

To carry on this work further, additional polymers were selected for analysis to study the abilities of polymers containing different types of functional groups to take up ammonium and bifluoride ions. We choose polyvinyl acetate (PVAC) and polyvinyl pyrrolidone (PVP) as polymers containing carbonyl groups, and polyethylene imine (PEI) and the shorter tetraethylenepentamine (TEPA) as polymers containing amine groups. We also chose to investigate molecules containing hydroxyl groups as OH^…^F hydrogen bonding interactions could be possible. Our initial tests with polyvinyl alcohol (PVA) failed, as the molecule was insoluble in the solvent system employed. However, after interesting tests with xylitol we also decided to test larger saccharide molecules, such as maltodextrin.

In this report, we present the one-pot syntheses of four polymer matrices and two smaller molecules containing coordinated bifluoride or fluoride ions. We first show how it is possible to use a standard fluoride sensitive electrode to measure bifluoride in solvents. Then, we present characterizations of the four new compounds and preliminary tests on these polymers as electrolytes in room temperature fluoride ion batteries along with their electrochemical performance.

## 2. Results

### 2.1. Measuring Bifluoride in Solvent: An Approach Based on a Standard Fluoride Sensitive Electrode

A critical component of testing fluoride ion-containing batteries is the determination of how many F^−^ ions are present in the electrolyte. Unfortunately, no sensors currently exist that can measure fluoride ion content in non-aqueous solution, or which can measure bifluoride ions at all. Therefore, as a first step, we used the following approach as a calibration to estimate fluoride content for further studies in solvent:

For investigations, the bifluoride ion, F_2_H^−^, must be split, which can be achieved by shifting the equilibrium F_2_H^−^ ⇄ F^−^ + HF ⇄ 2F^−^ + H^+^. As most electrolytes are slightly acidic, this change in equilibrium can be induced by adjusting the pH of the solution. Tests carried out showed that the most accurate measurement of F_2_H^−^ detection is reached at pH 7 (Table 1) and that at this point about 94% of the fluoride ion in the F_2_H-test solution could be measured. These measurements had to be performed in water as the fluoride sensitive electrode (FSE) reacts strongly on the presence of solvent or even trace of solvents in water, therefore it was not possible to measure these values in the electrolyte acetonitrile (MeCN) mixture.

We applied this approach to estimate fluoride contents of the different compounds. The detailed synthesis and naming of the compounds can be found in the Experimental Section. To determine fluoride content in the compounds, 0.1 g of material was solved in 4 mL water and the pH was adjusted to about pH 7 using sodium hydroxide. The solutions were then increased to 5 mL, with the exception of PVAC, which it was virtually insoluble under these conditions. The results are given in Table 2. We emphasize that these measurements give an approximation of the fluoride quantity.

### 2.2. Conductivity of the Fluoride Electrolytes and Reference Samples

The conductivity of polymer bifluoride matrices depended strongly upon the dissolution of the sample, due to the swelling effect of the polymer, which has a direct influence on the mobility of the ions being measured (Table 3). The conductivity was also dependent on the solvent used (see Supplementary Materials Figure S1). MeCN was chosen as a solvent, as it is the same solvent we used in the battery tests (described later). When preparing the solutions, 0.1 g of fluoride material in 4 mL of MeCN was used. The fluoride containing Xylitol compound (Xy-F) and the fluoride containing Maltodextrin compound (Malto-F) only dissolved slightly under these conditions, so measurements were carried out in a saturated solution of these compounds. As a reference for fluoride content and conductivity, we used Tetraglyme*NH_4_F_2_H (TGBF) from [22] which was dissolved in MeCN in the same manner. Previous studies reported that a saturated solution of KF in DMSO contained 8 mg of fluoride [25].

**Figure 2:**
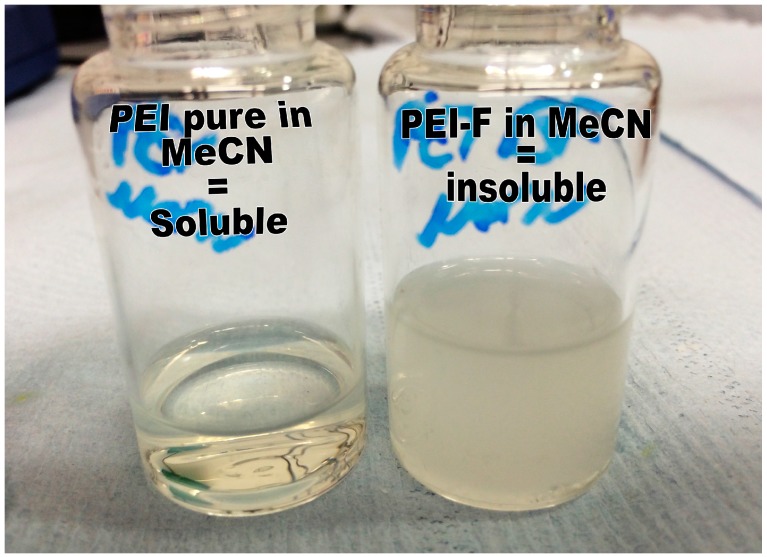
Change in physical properties of NH_4_F after reaction with PEI.

Both of the amine-containing compounds, PEI and TEPA, formed, in aqueous solution, a protonated polycation [26], and as side-reactions might occur, the values reported might not represent the actual fluoride concentration. Fluoride containing PEI polymer (PEI-F) also showed an unexpected behavior, whereby PEI was completely soluble in MeCN, while the product PEI-F showed almost no dissolution (Figure 2).

PVAC was not very soluble in water, so higher volumes of solvent were required. Regardless of this, the fluoride containing PVAC polymer (PVAC-F), as well as PVP-F, showed higher fluoride contents than the previously used tetraglyme ammonium bifluoride [22], while PVP-F also showed also better conductivity than the fluoride containing polymer reported in the publications before [22]. As these measurements were carried out at high dissolution, the values obtained do not indicate the maximum conductivity possible. Titrations can be carried out using different solvents and concentrations to determine the optimum conductivity and solvent systems for further battery tests. 

The most intriguing result obtained was that of the high conductivity measured of both Xy-F and Malto-F after filtration, since almost no dissolution of the compounds during the reaction could be observed.

### 2.3. NMR and IR Spectroscopic Results

**Figure 3:**
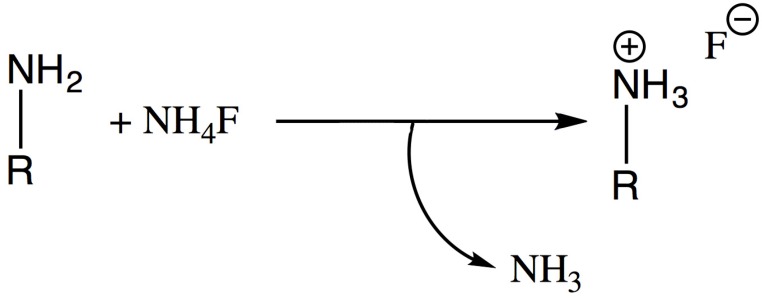
Reaction of ammonium fluoride with amine-containing polymers.

^19^F NMR spectroscopic measurements showed the typical fluoride doublet resonance at around −150 ppm for PVAC-F, PVP-F and Xylitol-F [27,28,29]. Malto-F in CD_3_CN also showed a similar resonance at −107 ppm, which is presumably attributable to F^−^ ions [30]. PEI and TEPA also showed the presence of F^−^ ions. We assume that a partial substitution of the secondary R–NH_2_ groups to form R–NH_3_^+^•F^−^ had taken place, which could explain the presence of F^−^, and not F_2_H^−^ as expected (Figure 3, see also Experimental Section for details of NH_3_ evolution).

**Figure 4:**
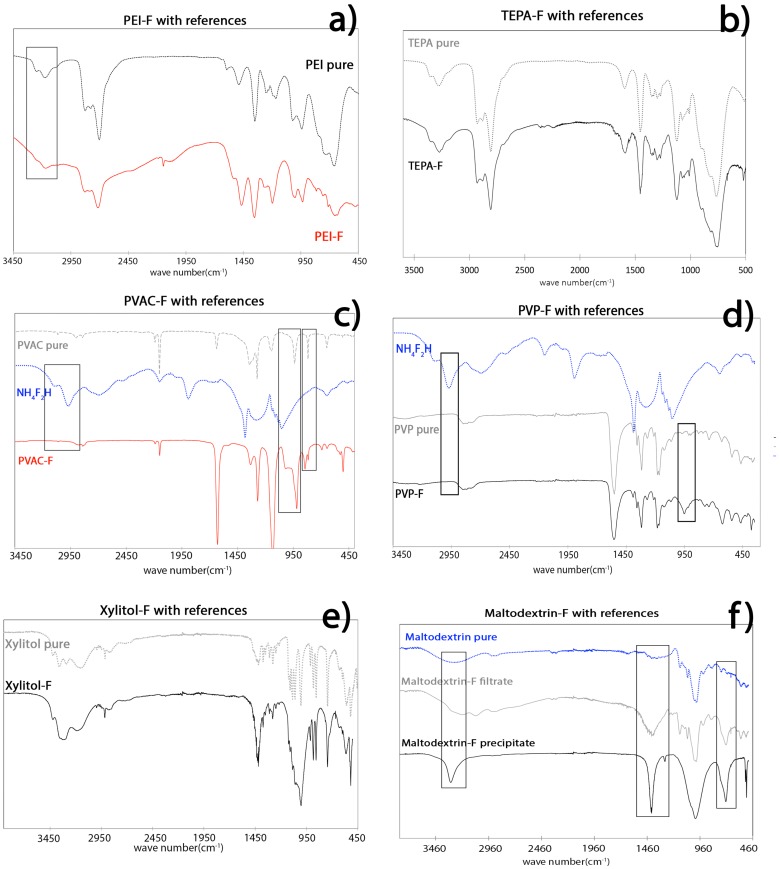
IR spectra of: TEPA-F (**a**); PEI-F (**b**); PVAC-F (**c**); PVP-F (**d**); Xy-F (**e**); and Malto-F (**f**).

For IR spectroscopic analyses, few changes to the spectra were expected as no “chemical bonds” were formed in the large polymer matrix, and only a few peak shifts could be observed between the spectra of the matrix and complexed product [31]. Furthermore, the quantity of hydrogen-bonded ammonium ions appeared to be less than expected, so no dramatic changes from the native polymer to the product formed were expected. The IR spectra of PEI-F and TEPA showed no obvious changes (Figure 4a,b). The same was also true for PEI-F, where the spectra showed very few differences between the product and the original trace of the native polyethylene amine molecule, other than the typical vibration of NH_4_F no longer being visible. One difference that was observed was the broadening of the band at around 3200 cm^−1^ [32], which could possibly be attributed to a change in the N–H vibration upon coordination to F^−^. There were also small changes in the fingerprint region, especially in the intensity of the bands.

The IR spectra of PVP-F and PVAC-F showed only small changes upon formation of the products, where the bands observed could be largely attributed to the polymer matrix (Figure 4c,d). In the case of PVAC-F, new bands appeared at 1000 cm^−1^ and at 900 cm^−1^, and similar to the previously reported observations for PEG-compounds, the band corresponding to the N–H vibrations disappeared. In the case of PVP-F, a very small peak at 2934 cm^−1^ and a double peak at 700 cm^−1^ appeared, in addition to broadening of the band at 950 cm^−1^.

When looking at the spectrum of Xy-F, no significant changes could be observed, other than a general broadening of the bands. The spectrum of Malto-F showed observable changes, however, where, in particular, the non-soluble Malto-F-precipitate showed a shift of the wider hydroxyl band at around 3353 cm^−1^ towards 3100 cm^−1^ and the formation of an additional band at 726 cm^−1^ (Figure 4e,f). We are unsure if these changes are an effect of N–H or F–OH bonding interactions. The fingerprint region showed a large band at 1447 cm^−1^, which is commonly attributed to a combination of OCH and CCH vibrations [33]. Similar to the Malto-F-precipitate, a new peak at 720 cm^−1^ appeared in the spectrum of Malto-F-filtrate, which can be attributed to the C–O and C–C vibrations of the carbohydrate group [33].

### 2.4. Characterization with X-ray Diffraction

In the case of PVP and PVAC, no structural information could be inferred from the PXRD spectra collected, and only the large signals of the polymers were observed (see Supplementary Materials Figure S2). 

PXRD can be helpful to determine if the product is pure and if an incorporation of the fluoride salt was achieved: sometimes the remaining starting product can be observed and a second recrystallization step is needed. However, the spectrum of Xy-F showed many small changes, which indicates that a structural change had occurred in the crystalline structure of the polymer. Similarly, the yellow precipitate of Malto-F showed the appearance of new peaks in the broad spectra of the maltodextrin matrix, indicating that some form of crystalline compound had formed. These small peaks were due to the phases of NH_4_BF_4_ [34] and NH_4_SiF_6_ [35], which were initially thought to be introduced to the sample from the glass reaction vessel used. When the reaction was repeated in plastic or Teflon vessels, however, these peaks were still observed, so it is most likely that the maltodextrin precursor contained a borosilicate impurity, which could be observed by energy-dispersive X-ray (EDX) spectroscopy of the starting materials. This issue of impurity could be overcome by recrystallization of the starting materials prior to their use (see Supplementary Materials Figure S3).

### 2.5. Optical Properties

**Figure 5:**
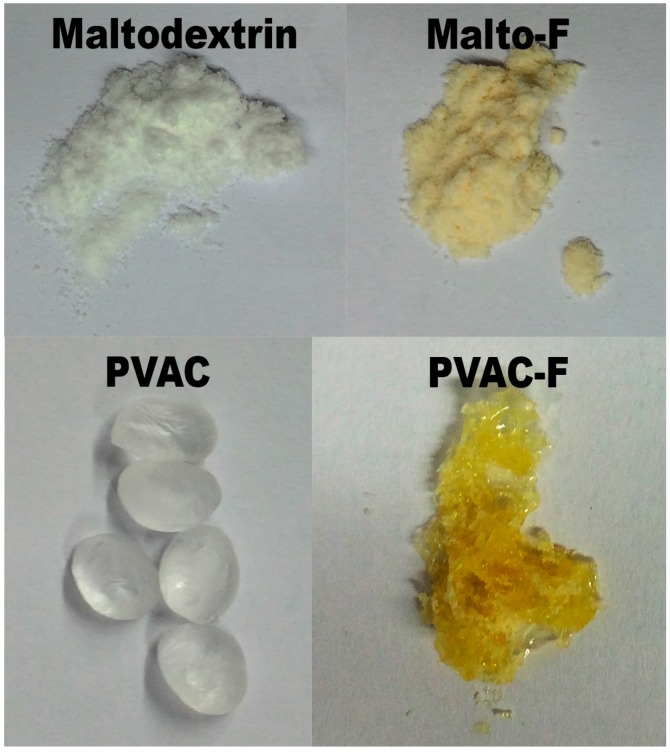
Color changes of Malto-F (soluble filtrate) and PVAC-F after reaction.

The most intriguing feature observed for these polymeric products was the yellow color of the compounds prepared (Figure 5).

These results were likely due to the presence of hydrogen bonding and the subsequent bathochromic shift. Bathochromic shifts are typical upon the coordination of anions [36] and it has been previously observed that the coordination of fluoride ions can generate such shifts [16,37]. Furthermore, similar effects were reported in examples of ammonium ion and ammonia sensing, whereby the coordination of ammonium also generated such a bathochromic shift [38,39,40,41]. To check if the ammonium ion was responsible for this color change, the reactions were repeated with NH_4_Cl in place of NH_4_F. For both PVAC and PVP, no color change could be observed, so we hypothesized that the color change observed was due to fluoride/bifluoride coordination to the polymers [37,42].

### 2.6. Characterization Summary

In summary, we conclude that, in the cases of PVAC and PVP, complexation of polymers by NH_4_F_2_H has occurred. Due to the low conductivity and fluoride concentration, as well as the presence of N atoms in the materials prepared (see Supplementary Materials Figure S3), we propose that NH_4_F_2_H has been incorporated into the polymer.

In the case of polymers containing PEI and TEPA, we propose that a partial protonation of the -NH_2_ group to -NH_3_F- may have occurred. There is also the possibility that there are free NH_4_^+^ ions in the molecule. Xy-F and Malto-F also show the presence of N atoms, which supports the thesis that both ammonium and fluoride ions have been introduced to the polymer matrices.

## 3. Discussion

### 3.1. Electrochemical Stability and Impedance Spectroscopy

Cyclic voltammetry (CV) and linear sweep voltammetry (LSV) studies were performed to test the electrochemical stability of the compounds and to make sure that no unwanted side-reactions would occur if the fluoride-containing polymers were subjected to a change in voltage. Neither ammonium fluoride and bifluoride or the polymer matrices alone showed any CV signals or special features, and therefore it was assumed that the new compounds would also not show any activity upon changes in voltage. The experiments were performed in MeCN to keep the conditions as similar as possible to those that would later be used in battery tests and applications, therefore PEI-F and TEPA-F were measured in pure state. The stability of the polymers against the Ag reference was observed to be identical of pure MeCN. (See Supplementary Materials Figure S4).

**Figure 6:**
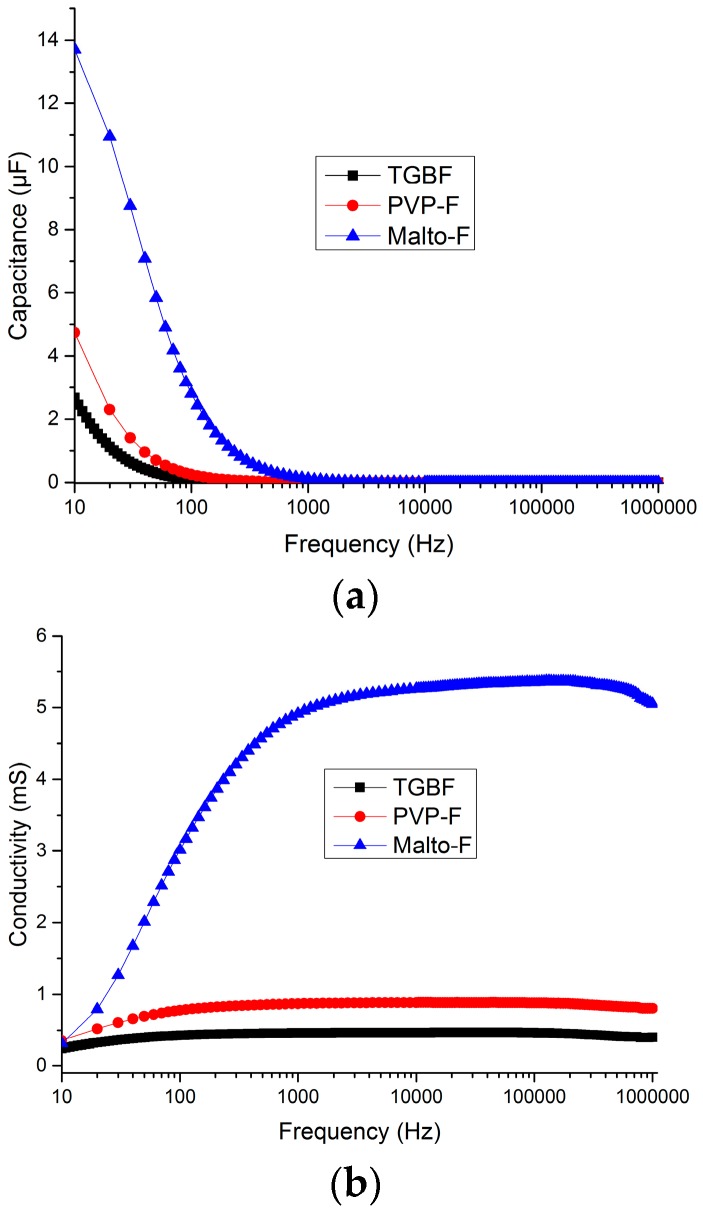
Frequency dependent conductivity spectra and frequency dependent capacitance spectra of compounds TGBF, PVP-F and Malto-F. (**a**) Capacitance; (**b**) Conductivity.

Subsequently, the electrochemical impedance spectra of the two compounds that showed the highest conductivity (PVP-F and Malto-F) were measured and compared to the already known electrolyte Tetraglmye*NH_4_F_2_H (Figure 6). 

The impedance spectra were measured from high frequency (10^6^ Hz) to low frequency (10 Hz), and the conductivity values were calculated by taking the inverse of the real component of the impedance. At frequencies above 10^3^ Hz, the spectra are nearly frequency-independent (Malto-F demonstrates a slight decrease with decreasing frequency) and no features are observed. Both PVP-F and Malto-F show higher conductivity values than the reference electrolyte, however the conductivity of Malto-F is observed to over five times higher than that of PVP-F in this frequency range. It should be noted that the conductivity values were not normalized for the cell geometry, and are given in milli Siemens (mS). At lower frequencies (below 10^3^ Hz) the conductivity drops. This corresponds to an increase in the capacitance at these frequencies (Figure 5a), and can be attributed to the build up of charged species at the electrode interface at low frequencies approaching the DC case.

### 3.2. Preliminary Tests of Electrolytes in RT-FIB

The electrolytes were tested in simple fluoride ion test cells. The cathode was prepared from BiF_3_, Carbon Black and Carboxy-Metylcellulosis (CMC), and a separator comprised of either cotton or a Whatman (glasswool) filter. The anode consisted of an Mg-band that was cut into small pieces and sanded before use. It was also important that solvents were used that would not damage the anode, therefore MeOH, EtOH, and H_2_O were not employed in these experiments. Although only the surface of the cathode and anode would come in direct contact with the electrolytes, the total bulk of these materials were used in calculations [24]. The curves generally showed variations in their shape and performance, on account of the batteries being made and assembled by hand. We therefore chose to omit the worst and best curves and instead select a curve to study that reflected the average performance of the battery.

It is known that pure mixtures of electrolyte in MeCN can dry out easily [21,22] and, as a result, lengthy experiments are not possible. However, we decided against the use of higher boiling solvents or additives in order to avoid the addition of further parameters to the system and any side effects that might arise. The fact that the discharge curves obtained were not completely smooth and showed irregularities, especially at the end of the discharge, are most likely a result of the drying out of the electrolyte, however. Furthermore only the first discharge was investigated, as it was not possible to study multiple cycles using Mg metals due to the formation of an insoluble and dense MgF_2_ layer. Currently, no better anode material exists, however, which would avoid these complications with carrying out measurements, and research is ongoing to address this issue. Furthermore, it was not possible to study the properties of PEI and TEPA in these battery systems, as the compounds on their own were too reactive and destroyed the separators and we were unable to find an appropriate solvent system that did not result in coagulation of the materials. Therefore, no discharge could be observed.

In general, all four of the electrolytes tested (PVAC-F, PVP-F, Malto-F and Xy-F) showed an open circuit voltage (OCV) of 1.3 V–1.7 V, which is higher than that observed for the reported PEG-based electrolytes. This OCV value was followed by a sharp drop in voltage, which seemed to be a property of the cathode material and not of the electrolyte. Further tests in which the carbon black was replaced by carbon fibers did not show this large drop (see Supplementary Materials Figure S5).

**Figure 7:**
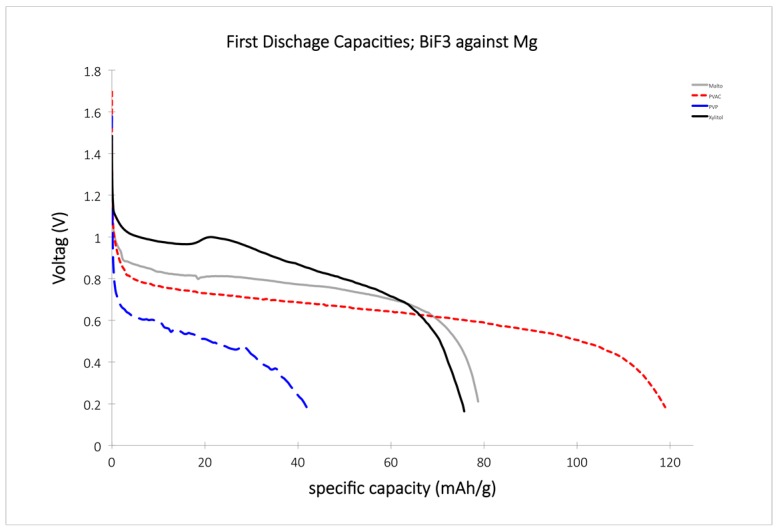
Discharge curves for the various fluoride-doped polymer compounds.

PVAC showed an OCV of about 1.7 V, which is 1 V below the theoretical OCV of 2.7 V for the system BiF_3_ against Mg, and a discharge plateau at about 0.7 V. The discharge curve was smooth and showed a total gravimetric capacity of about 120 mAh/g (Figure 7).

Xy-F and Malto-F performed very similarly to each other, and furthermore, Malto-F showed the highest discharge plateau of all the polymers studied with a voltage of about 1 V. PVP, even with its high fluoride content, did not perform as well, however, with its discharge plateau at around 0.5 V, and it only reached a capacity of about 40 mAh/g.

**Figure 8:**
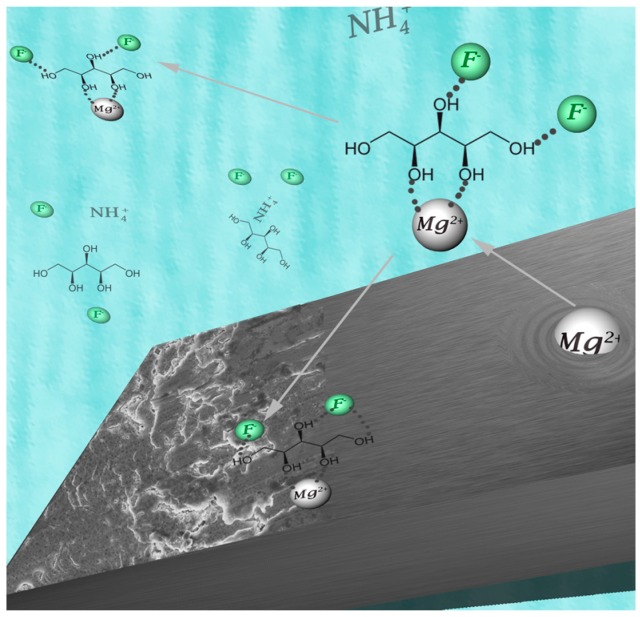
Possible formation of Mg^2+^-based complexes with Xy-F in both the electrolyte and at the anode surface, which could cause issues with the battery performance.

Excluding PVP-F, all of these new electrolytes appeared to function slightly better than the known PEG-based electrolytes. Tests on the anode of the batteries using scanning electron microscopy (SEM) and EDX spectroscopy, however, showed the formation of an MgF_2_ layer and, for the Xy-F electrolyte, larger amount of C and O. Traces of Mg were also detected at the cathode. We therefore hypothesize that the sugar-based electrolytes were more susceptible to the formation of Mg^2+^ complexes at the anode [43], which may decompose at the anode or remain in the electrolyte (Figure 8). In the case that this issue with dissolution of the anode remains, even once a new anodic material is realized, a protection layer on the anode would be necessary. Nevertheless, these electrolytes, especially the sugar-based electrolytes, offer an interesting approach for future and sustainable fluoride battery applications.

## 4. Materials and Methods

### 4.1. Materials

The starting materials were purchased from VWR or Sigma-Aldrich and were not purified before syntheses. The syntheses were carried out under normal conditions in air. 

As a general procedure, the fluoride salt and polymer matrix were mixed in acetonitril (MeCN) and refluxed for several hours. NH_4_F or NH_4_F_2_H are not typically soluble in MeCN, while some polymers showed only limited dissolution. Therefore, large excesses were used to ensure the best possible reaction outcomes. The unreacted compounds were filtered off at the end of the reactions.

Infrared (IR) spectra were recorded on a Perkin Elmer Spectrum Two. Powder X-ray diffraction (PXRD) analyses were measured on a STOE Stadip-2 with a copper source in transmission mode. Nuclear magnetic resonance (NMR) spectra were recorder on a 400-MHz Bruker 300 Ultrashield, with 64 scans and from −100 to −280 ppm. Fluoride and conductivity measurements were performed on a Mettler Toledo Sven Excellence with the appropriate electrodes. Cyclic voltammograms were measured using a Dropsene y-400 with screen-printed electrodes, carbon as the working anode, silver as a pseudo-reference and silver as the center electrode. Test cells were made in standard Teflon swagelock-construction and in home-made multi cells, the measurement were performed on a ARBIN with a discharge current of 0.01 mA and cut-off voltage of 0.1 V. Cyclovoltammerty and linear sweep voltammetry measurement were performed on a yStat 400 Dropsense. To determine the conductivity of the samples, electrochemical impedance spectroscopy was performed with a potentiostat equipped with a frequency analyzer (PGSTAT302N from Metrohm Autolab, Utrecht, The Netherlands) and the corresponding software. A three-electrode cell was used. The working electrode was an indium-tin-oxide coated on a glass substrate, the counter electrode was a Pt mesh and the reference electrode was Ag wire. The cell geometry was the same for each sample. Spectra were recorded in a frequency range between 1 Hz and 10^6^ Hz by applying a small signal V_AC_ with an amplitude of 10 mV. 

### 4.2. Experimental Section

Synthesis of a F_2_H^−^ doped PVAC Matrix (PVAC-F): 

PVAC granules (2 g, 0.04 mmol, M_W_ = 50,000 g/mol) and NH_4_F (0.37 g massive excess, about 10 mmol) or NH_4_F_2_H (0.62 g, massive excess, about 10 mmol) were dissolved in MeCN (50 mL). The solution was stirred at 85 °C for 5 h. After cooling down slowly, the mixture was stirred overnight at room temperature. A bright yellow clear solution was obtained and the solution was filtered to remove excess NH_4_F/NH_4_F_2_H residues. The solvent was removed slowly under reduced pressure, until a pale yellow solid was obtained and the residue dried in a vacuum oven at 70 °C or on a heating plate at 100 °C in a glovebox.

^19^F-NMR (CD_3_CN): δ (ppm) = −151.64, −151.59

IR (cm^−1^) (from electrolyte): 2280 (m), 1730 (s), 1445 (w), 1375 (s), 1230 (s), 1125 (w), 1020 (s), 980 (m), 945 (w), 820 (w), 765 (w), 680 (w), 640 (m)

Conductivity (100 mg in 4 mL MeCN): 229 µS/cm

Synthesis of a F_2_H^−^ doped PVP Matrix (PVP-F):

PVP (1.5 g, 0.037 mmol, M_W_ = 40,000 g/mol) and NH_4_F (0.37 g massive excess, about 10 mmol) or NH_4_F_2_H (0.62 g, massive excess, about 10 mmol) were dissolved in MeOH (40 mL). The solution was stirred at 85 °C for 3 h. After cooling down slowly, the mixture was stirred overnight at room temperature. A pale yellow clear solution was obtained. For further preparation of the electrolyte, a portion of the reaction mixture (6 mL) was added to MeCN (20 mL). The mixture was refluxed at 100 °C while stirring for at least 2 h. After cooling to room temperature, the solution was filtered and solvents were removed slowly under reduced pressure and the residue was dried in a vacuum oven at 70 °C or on a heating plate at 100 °C in a glovebox.

^19^F-NMR (CD_3_CN): δ (ppm) = −151.48, −151.43

IR (cm^−1^) (from residue): 2965 (w), 1660 (s), 1500 (w), 1465 (m), 1430 (s), 1375 (w), 1305 (w), 1285 (s), 1265 (s), 1230 (w), 1180 (w), 1070 (m), 875 (w), 840 (w), 730 (m), 660 (m), 570 (m), 490 (m)

Conductivity (100 mg in 4 mL MeCN): 417 µS/cm

Synthesis of an electrolyte based on TEPA with MeCN (TEPA-F):

Direct mixing of NH_4_F (0.3 g, 80 mmol) in liquid TEPA (1.5 mL, 10 mmol) produced, after 1 h, a thick gel-like substance, which was insoluble in common organic solvent and difficult to purify, therefore another approach was chosen for the synthesis of TEPA-F.

TEPA (2 mL, 10 mmol) and NH_4_F (0.1 g, 20 mmol) were dissolved in MeCN (20 mL). This suspension was heated to 90 °C for 2 h. A crystalline, very hygroscopic precipitate was formed, which, after cooling of the reaction mixture to room temperature, could be removed by filtration. The removal of excess solvent led to the isolation of a pale yellow viscous residue.

^19^F-NMR (CD_3_CN): δ (ppm) = −121

IR (cm^−1^) (from residue): 3223 (broad), 3075 (w), 2964 (s), 2873 (m), 2667 (w), 2637 (w), 2543 (m), 2392 (w), 1190 (s), 1066 (broad), 803–703 (w, broad)

Conductivity (0.1 g in 4 mL MeCN): 120 µS/cm (not completely soluble, a white precipitate formed)

Synthesis of a F_2_H^−^ doped PEI Matrix (PEI-F):

Ground NH_4_F (0.2 g, massive excess, 54 mmol) dissolved in MeCN (10 mL) and branched PEI (2 mL, M_W_ ≈ 800 g/mol, 2.5 mmol) in MeCN (30 mL) were combined. The mixture was heated to 100 °C for 5 h, then cooled to room temperature. Subsequent filtering was necessary to remove unreacted NH_4_F. After removing the solvent under reduced pressure, the milky solution became a clear yellow resinous liquid. Further drying at 80 °C and 10 mbar pressure led to an almost solid residue.

^19^F-NMR (CD_3_CN): δ (ppm) = −106.5 (broad)

IR (cm^−1^) (from residue): 3250 (m), 2930 (w), 2840 (w), 2750 (s), 2250 (m), 1625 (w), 1570 (s), 1465 (s), 1360 (w), 1285 (s), 1110 (m), 1035 (m), 930 (w), 855 (w), 820 (w), 770 (w)

Conductivity (100 mg in 4 mL MeCN): 176 µS/cm (forms a white precipitate, incomplete dissolution)

Synthesis of a F_2_H^−^ doped Xylitol Matrix (Xy-F):

Xylitol (1.5 g, 9.8 mmol) and NH_4_F_2_H (0.5 g, 80 mmol) were ground by mortar and pestle and added to MeCN (30 mL). The suspension was stirred and heated to 120 °C for at least 2 h, over which time an almost clear solution was formed. After cooling to room temperature, the suspension was stirred overnight. After filtering, a white precipitate was obtained, which X-ray diffraction (XRD) analysis showed to be primarily unreacted starting materials. The liquid was evaporated under pressure and a white flaky powder obtained.

^19^F-NMR (CD_3_CN): δ (ppm) = −151.74, −151.69

IR (cm^−1^) (from residue): 3320 (s), 2930 (w), 1430 (s), 1300 (w), 1000 (s), 875 (w), 750 (m), 570 (w), 520 (m)

Conductivity of the filtrate (100 mg in 4 mL MeCN): 5927 µS/cm

Synthesis of a F_2_H^−^ doped Maltodextrin Matrix (Malto-F):

Maltodextrin (1.2 g, 7 mmol) and NH_4_F_2_H (0.9 g, 15 mmol) were ground by mortar and pestle and added to MeCN (100 mL). The suspension was stirred and heated to reflux for at least 4 h. After cooling down to room temperature, a yellow-orange precipitate formed, which was removed by filtration. The precipitate was not soluble in MeCN. The filtrate was dried to obtain an off-white powder, which was readily soluble in MeCN and used for further analyses.

^19^F-NMR of the filtrate (CD_3_CN): δ (ppm) = −151.42, −151.46

Conductivity of precipitate (100 mg in 4 mL MeCN): 350 µS/cm (only slightly soluble)

Conductivity of filtrate: (≈80 mg in 4 mL MeCN): ≈6200 µS/cm (soluble)

### 4.3. NH_3_-Evolution

It was hypothesized that NH_3_ could be released during the reactions, in particular in the reaction of NH_4_F with amine groups (Figure 3).

**Figure 9:**
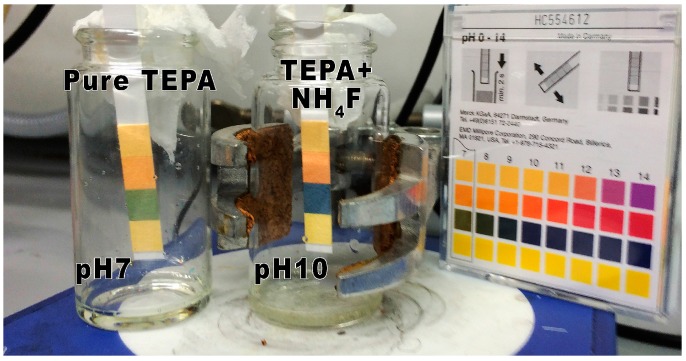
Simple NH_3_ detection test with pH paper.

The gas evolution of these reactions was monitored in two ways. Initially, tests using pH-paper showed that the simple stirring of NH_4_F in the presence of TEPA or PEI released a basic gas (Figure 9). A further test was also carried out, whereby the gas released was passed by gas-outlet into an aqueous solution of CuCl_2_. During the reaction the color of this solution changed from colorless to deep blue due to the formation of [Cu(NH_3_)_4_]Cl_2_ upon the evolution of NH_3_. (See Supplementary Materials Figure S6). For the other reactions, NH_3_ gas evolution could be observed, but only over longer reaction times with heating.

## 5. Conclusions

A range of polymers containing different functional groups was selected for use as the matrix for incorporation of fluoride and bifluoride anions using a variety of coordination motifs. Using a straightforward synthetic approach under mild conditions, ammonium fluoride or ammonium bifluoride were reacted with the five selected polymeric compounds. While the amine-containing polymer, PEI, and oligomer, TEPA, seemed to be directly protonated and could absorb pure fluoride ions, the other compounds consistently showed a transformation to the bifluoride ions. The fluoride quantity was measured using a work around strategy, since the current sensors employed cannot detect the bifluoride ion. For all compounds, characterizations were performed using IR and NMR spectroscopies, PXRD, CV, and conductivity measurements. A few selected compounds were also tested with impedance spectroscopy. The compounds prepared, excluding PEI and TEPA, which were too reactive, were prepared as electrolytes and used in preliminary room temperature fluoride ion battery tests. PVAC-F showed a long and smooth first discharge, and both saccharide-based electrolytes, Malto-F and Xy-F, showed a good discharge plateau. These materials are promising for further development in fluoride ion battery preparation and study. In summary, we propose a new matrix family for fluoride/bifluoride-containing electrolytes, and address the previously encountered issues of free fluoride chemistry in non-aqueous solvents.

## Figures and Tables

**Table 1:** Fluoride content dependence on changes in pH.

Theoretical Amount of F^−^ Present in the Test Solution (ppm)	pH	Measured F^−^ Amount (ppm)	Percentage of Monitored F^−^ (%)
1000	3.9	497	50
885	7.35	832	94
664	9.54	576	86

**Table 2:** Fluoride content and conductivity of the different compounds.

Electrolyte	F^−^ Concentration (ppm)
PVAC-F	19
PVP-F	69
PEI-F	68
TEPA-F	2.4
Xy-F Filtrate	3.7
Malto-F precipitate	14
Reference TGBF	12.5
Reference KF in DMSO (saturated)	8

**Table 3:** Fluoride content and conductivity of the different compounds (for the abbrevations also refer to experimental section).

Electrolyte	Conductivity µS/cm (0.1 mg in 4 mL MeCN)
PVAC-F	229
PVP-F	417
PEI-F	176 *
TEPA-F	120 *
Xy-F Filtrate	5972
Malto-F precipitate	350 *
Malto-F filtrate	6300
Reference TGBF	273
Reference PVCA	8
Reference PEI	41.5
Reference TEPA	65.5
Reference Xylitol	7.7 *
Reference Maltodextrin	10.3 *
Reference KF in DMSO (saturated)	8

* not completely soluble.

## Supplementary Materials

The following are available online at www.mdpi.com/1996-1944/9/12/965/s1. Figure S1: Conductivity depending on solvent; Figure S2: PXRD of PVP-F (with remaining starting product, before it was recrystallized) and PVAC-F; Figure S3: EDX-analysis of different electrolytes after synthesis and purification; Figure S4: Cyclovoltagrams of the different electrolytes and MeCN as reference; Figure S5: Discharge curve using carbon fiber fabric; Figure S6: Evolution of ammonia.
