# Supplementary material for: Simple One-Pot Syntheses and Characterizations of Free Fluoride- and Bifluoride-Containing Polymers Soluble in Non-Aqueous Solvents
Source: Materials (Basel). 2016 Nov 30;9(12):965. doi: 10.3390/ma9120965 (PMC5456975; doi:10.3390/ma9120965)
Supplement: Supplementary file 1 [file materials-09-00965-s001.pdf]

Supplementary Materials: Simple One-Pot Syntheses and Characterizations of Free Fluoride- and Bifluoride-Containing Polymers Soluble in Non-Aqueous Solvents

Dominik Steinle, Laura Friedrich, Nico Bevilacqua, Elizabeth von Hauff and Fabienne Gschwind

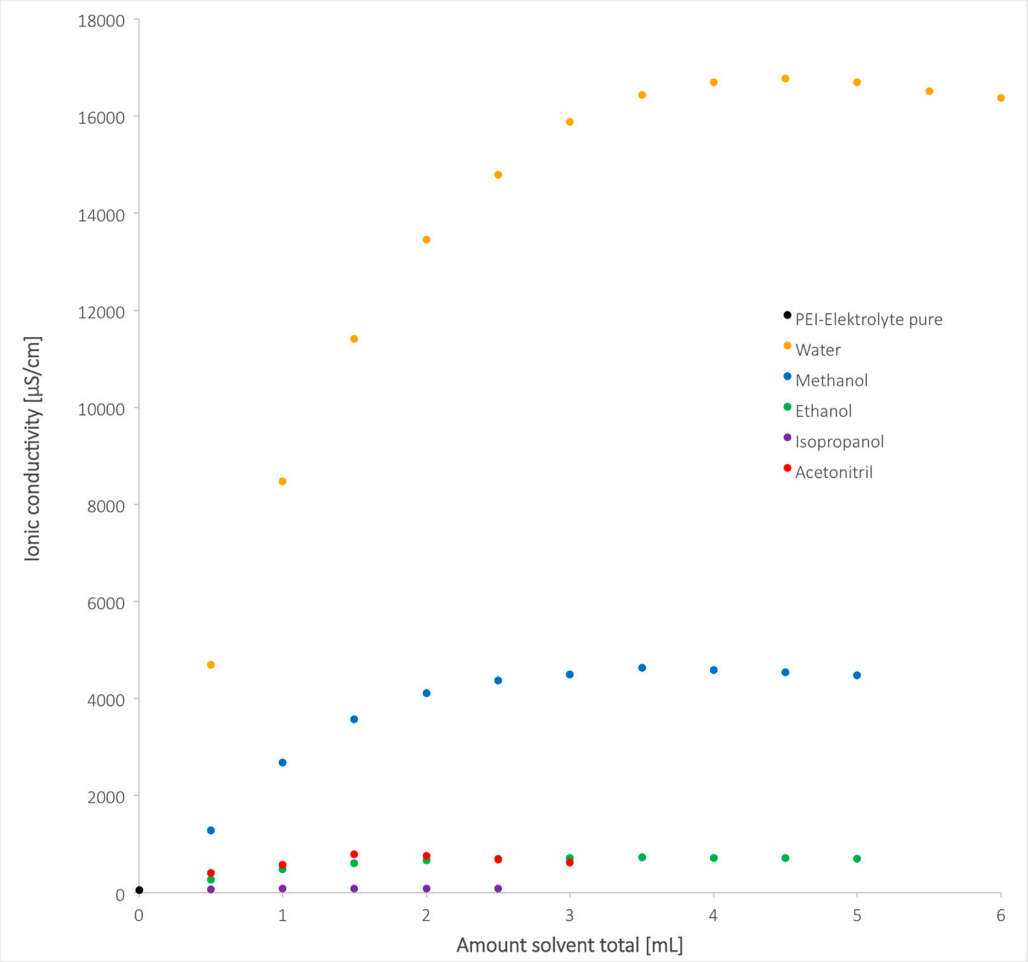

Figure S1. Conductivity depending on solvent.

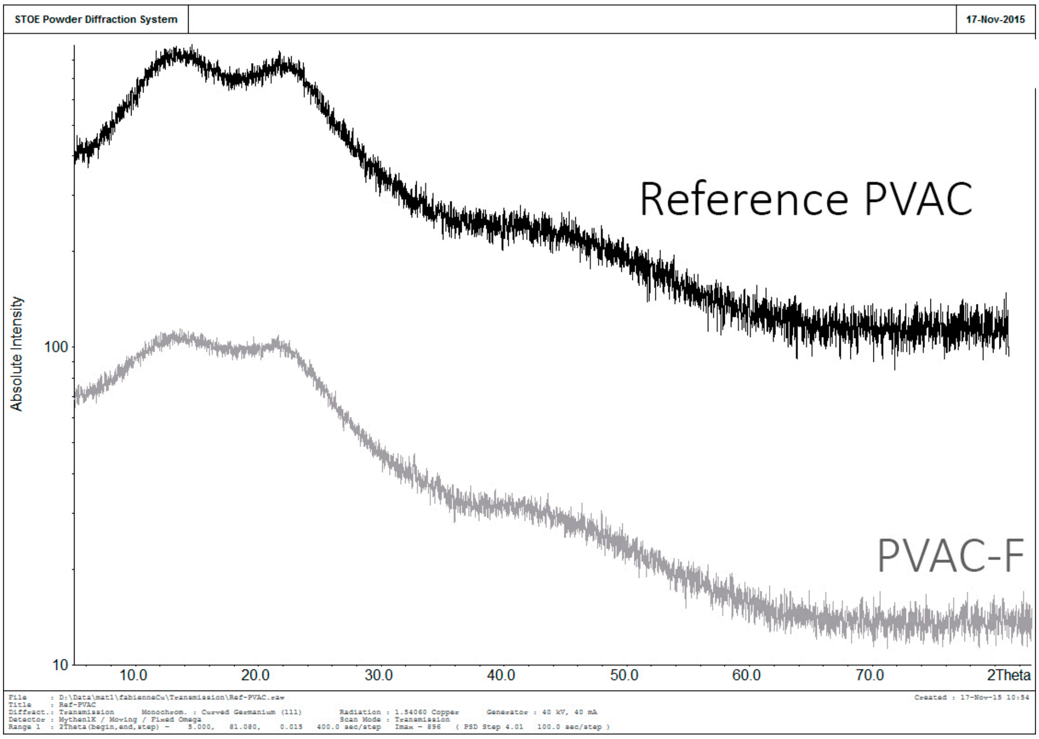

(**a**)

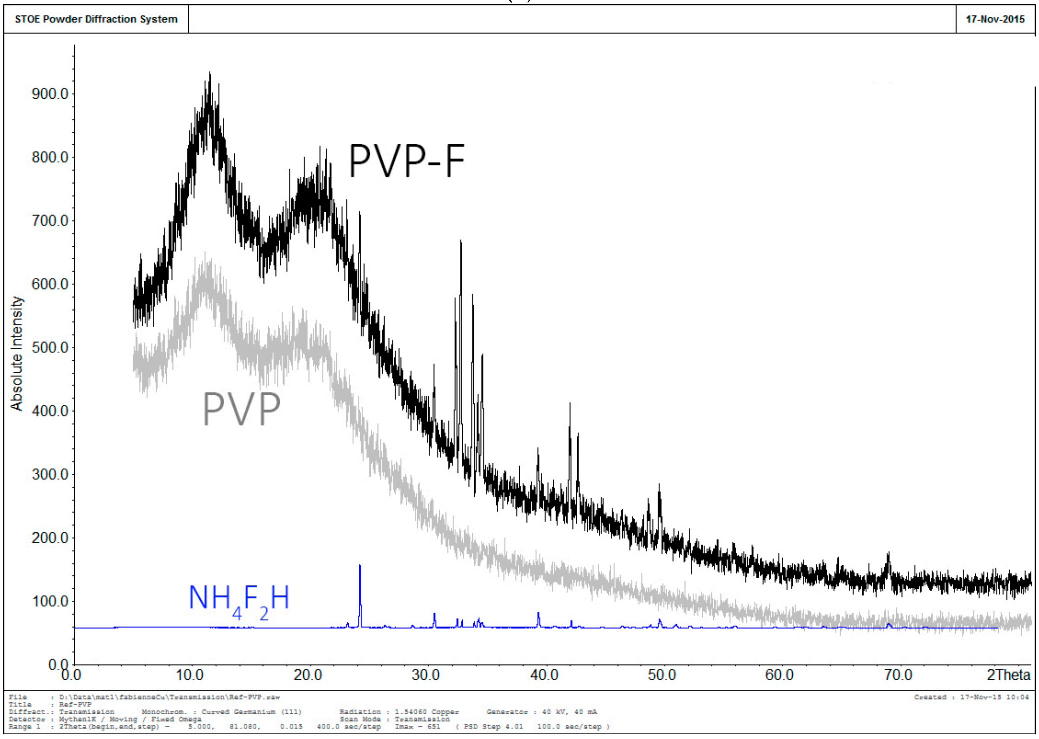

(**b**)

Figure S2. PXRD of PVP-F (with remaining starting product, before it was recrystallized) and PVAC-F. (**a**) PVCA with and without fluoride doping; (**b**) PVP pure polymer, PVP-F and starting material $NH_4F_2H_2$.

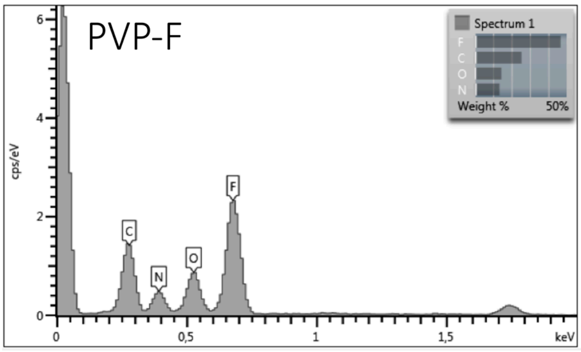

(**a**)

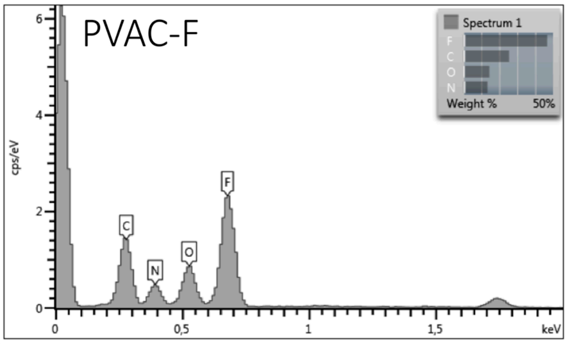

(**b**)

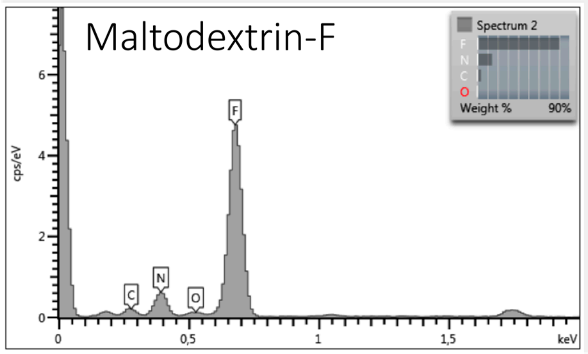

(**c**)

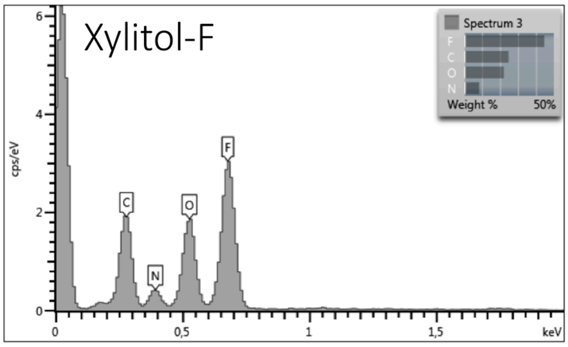

(**d**)

Figure S3. EDX-analysis of different electrolytes after synthesis and purification. (**a**) PVP-F; (**b**) PVAC-F; (**c**) Malto-F; (**d**) Xy-F [1].

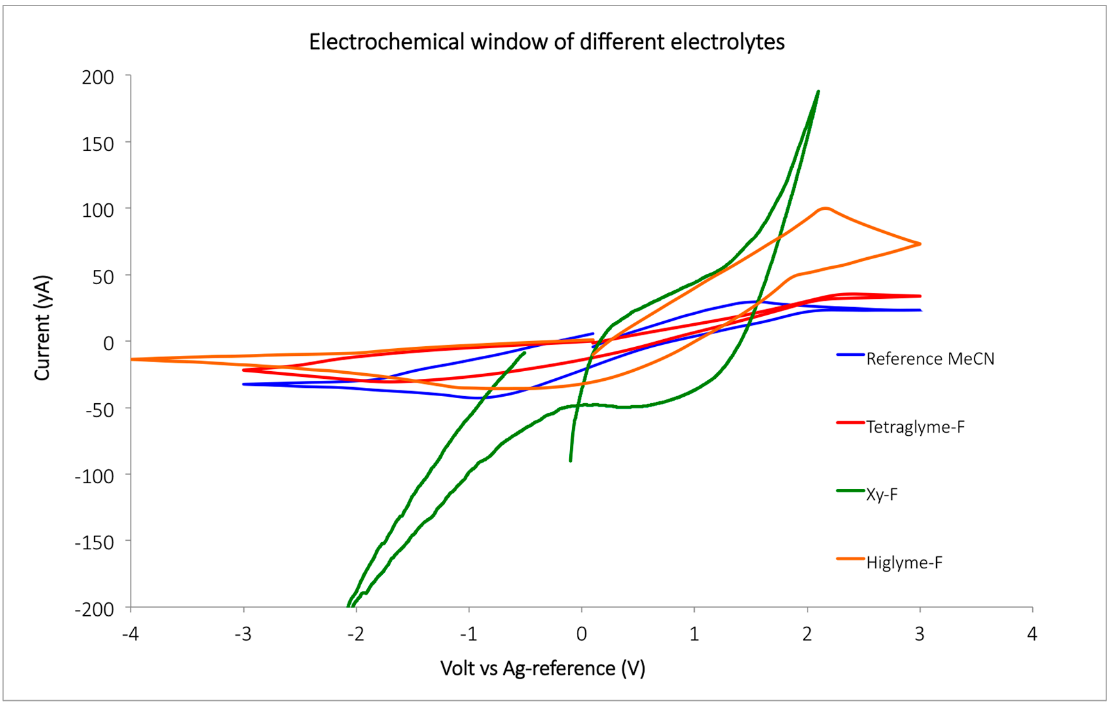

(**a**)

Figure S4. *Cont.*

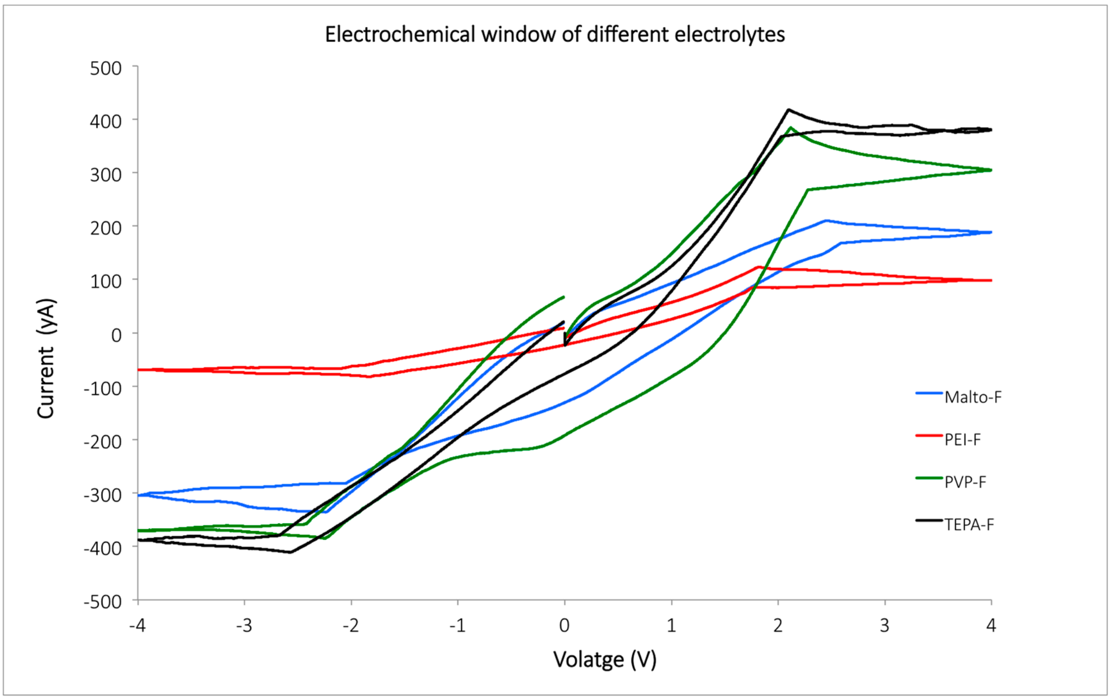

(**b**)

Figure S4. Cyclovoltagrams of the different electrolytes and MeCN as reference. (**a**) Reference MeCN, Reference TGBF, Xy-F and Reference Higlyme with bifluoride; (**b**) Malto-F, PEI-F, PVP-F, TEPA-F.

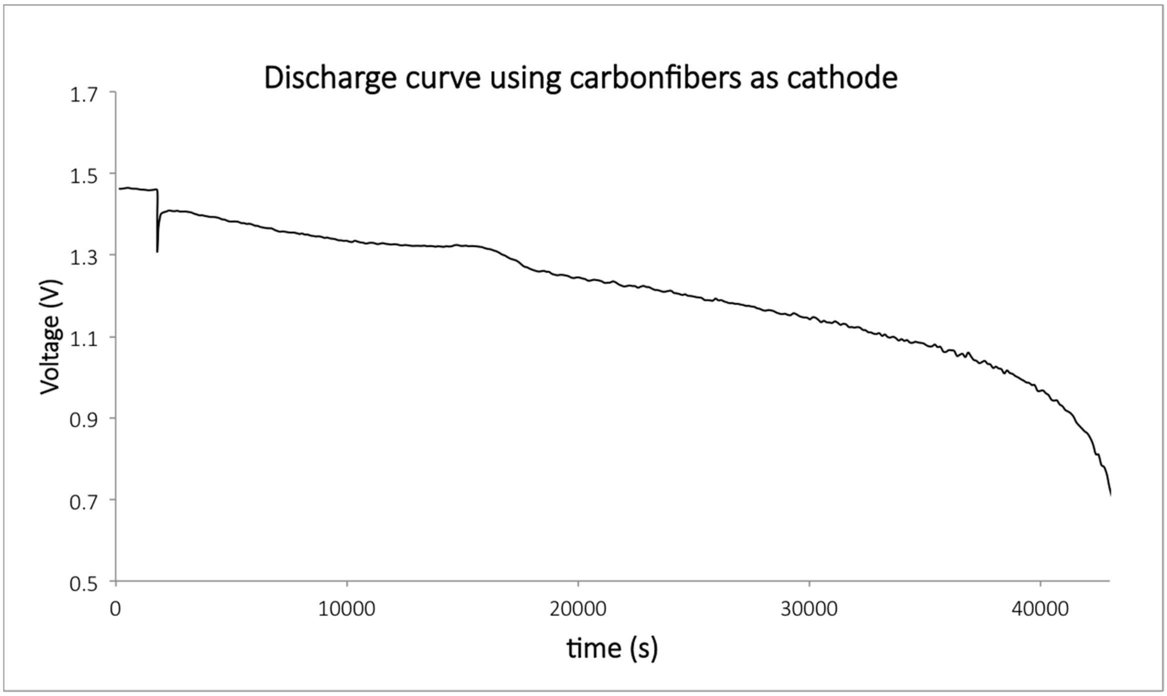

Figure S5. Carbon Fibers.

Discharge curve using carbon fiber fabric: Carbon fiber fabric (Kynol) was used as cathode material and coated with NH_4AlF_6, Mg was used as anode and Xy-F as electrolyte.

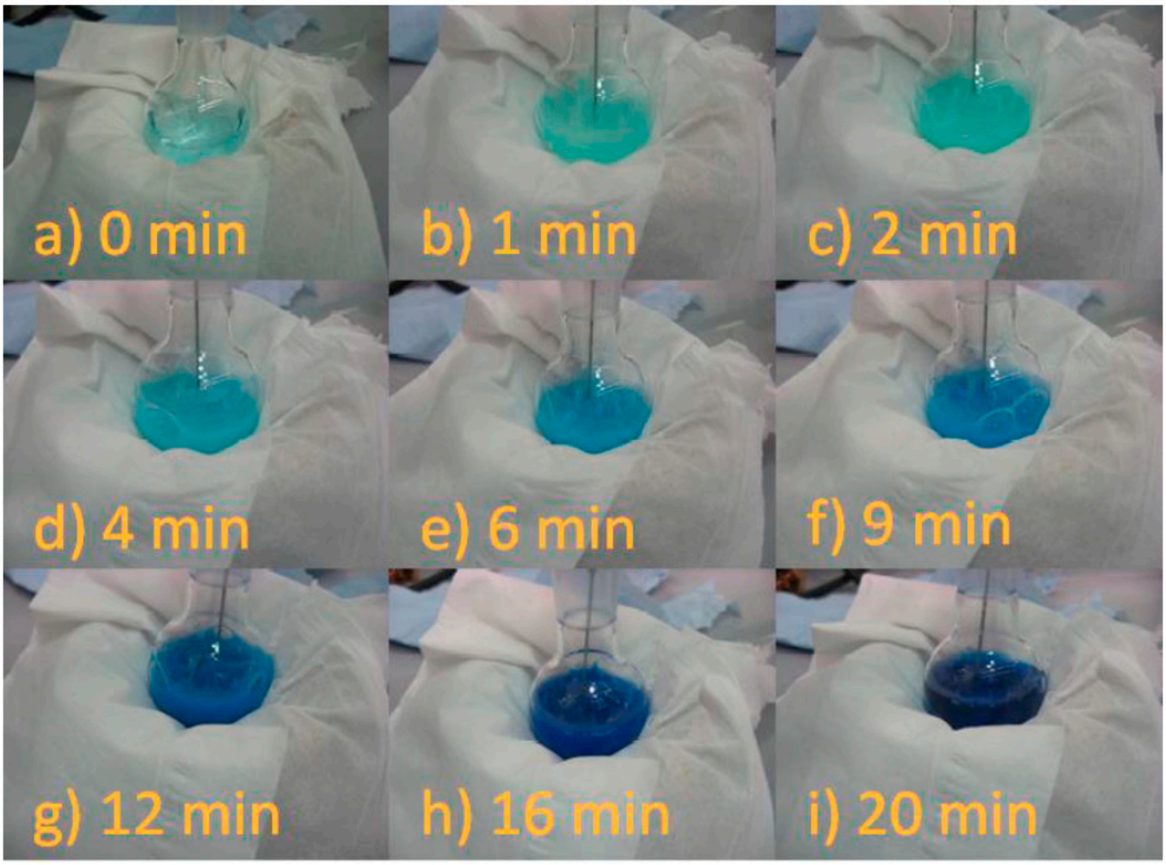

Figure S6. Evolution of ammonia: time-depending incoming of NH_3 evolution into a Cu(II)-containing solution, the formation of copper tetramine can be observed.

Reference

1. Vandi, L.-J.; Truss, R.; Veidt, M.; Rasch, R.; Heitzmann, M.T.; Paton, R. Fluorine Mobility During SEM-EDX Analysis: A Challenge for Characterizing Epoxy/Fluoropolymer Interfaces. *J. Phys. Chem. C* **2013**, *117*, 16933–16941.
